# Extracellular Vesicles Secreted by Adipose Tissue during Obesity and Type 2 Diabetes Mellitus Influence Reverse Cholesterol Transport-Related Gene Expression in Human Macrophages

**DOI:** 10.3390/ijms25126457

**Published:** 2024-06-12

**Authors:** Kseniia V. Dracheva, Irina A. Pobozheva, Kristina A. Anisimova, Aleksandra A. Panteleeva, Luiza A. Garaeva, Stanislav G. Balandov, Zarina M. Hamid, Dmitriy I. Vasilevsky, Sofya N. Pchelina, Valentina V. Miroshnikova

**Affiliations:** 1 Petersburg Nuclear Physics Institute Named by B.P. Konstantinov of National Research Centre “Kurchatov Institute”, 188300 Gatchina, Russia; fatal-ks@mail.ru (K.V.D.); perhaps_to_be@mail.ru (I.A.P.); aleksandra9122@mail.ru (A.A.P.); garaeve.luiz@yandex.ru (L.A.G.); sopchelina@hotmail.com (S.N.P.); 2 Department of Molecular-Genetic and Nanobiological Technologies, Scientific Research Center, Pavlov First Saint Petersburg State Medical University, 197022 St.-Petersburg, Russia; 3 Center for Surgical Treatment of Obesity and Metabolic Disorders, Pavlov First Saint Petersburg State Medical University, 197022 St.-Petersburg, Russia; anisimova-k-a@mail.ru (K.A.A.); stasbal@gmail.com (S.G.B.); zarina.hamid@yandex.ru (Z.M.H.); vasilevsky1969@gmail.com (D.I.V.); 4 Federal State Budgetary Research Institution “Institute of Experimental Medicine”, 197022 St.-Petersburg, Russia

**Keywords:** obesity, type 2 diabetes mellitus, cardiovascular disease, adipose tissue, extracellular vesicles, reverse cholesterol transport, macrophages

## Abstract

Obesity is a risk factor for type 2 diabetes mellitus (T2DM) and cardiovascular disease (CVD). Adipose tissue (AT) extracellular vesicles (EVs) could play a role in obesity and T2DM associated CVD progression via the influence of their specific cargo on gene expression in recipient cells. The aim of this work was to evaluate the effects of AT EVs of patients with obesity with/without T2DM on reverse cholesterol transport (RCT)-related gene expression in human monocyte-derived macrophages (MDMs) from healthy donors. AT EVs were obtained after ex vivo cultivation of visceral and subcutaneous AT (VAT and SAT, respectively). *ABCA1*, *ABCG1*, *PPARG*, *LXRβ (NR1H2)*, and *LXRα (NR1H3)* mRNA levels in MDMs as well as in origine AT were determined by a real-time PCR. T2DM VAT and SAT EVs induced *ABCG1* gene expression whereas *LXRα* and *PPARG* mRNA levels were simultaneously downregulated. *PPARG* mRNA levels also decreased in the presence of VAT EVs of obese patients without T2DM. In contrast *ABCA1* and *LXRβ* mRNA levels tended to increase with the addition of obese AT EVs. Thus, AT EVs can influence RCT gene expression in MDMs during obesity, and the effects are dependent on T2DM status.

## 1. Introduction

Obesity is a well-established risk factor for the development of concomitant type 2 diabetes mellitus (T2DM), atherosclerosis, and subsequent cardiovascular complications [1,2]. Cardiovascular diseases (CVDs) are the main cause of mortality in T2DM persons [3,4]. T2DM duration and a late onset of glycemic control are associated with an increased incidence and severity of CVD [3,4,5]. The mechanisms by which obesity and T2DM facilitate atherosclerotic processes remain poorly understood. Adipose tissue (AT) inflammation, AT secretome with imbalanced production of adipocytokines, and extracellular vesicles (EVs, exosomes) are discussed as potential links between obesity and its comorbidities [6].

EVs are membrane particles with sizes ranging from 40 to 160 nm in diameter that play an important role in cellular communications [7]. EVs transport different bioactive compounds such as functional proteins, including several adipokines in the case of AT, metabolites, and nucleic acids, including microRNA [8,9]. Additionally, AT EVs are enriched with lipids—fatty acids, triacylglycerols (TAGs), and cholesterol [10]. The higher plasma concentration of EVs in obesity and T2DM may be associated with alterations in EV cargo [11,12]. The latest data point suggests that the biogenesis of AT EVs is expected to be disturbed in obesity [13,14]. Recently, we described morphological changes in AT EVs during obesity [13]. Additionally, we and others showed that AT EV miRNA composition may also differ in obese patients and potentially could result in the dysregulation of gene expression linked to insulin resistance and lipid accumulation [15,16].

It is believed now that AT EVs can impact locally as well as influence distal tissues participating in CVD progression at different steps, such as endothelial dysfunction, lipid deposition, plaque formation, and rupture [6]. Moreover, obesity is associated with macrophage accumulation in ATs and the interaction of adipocytes with macrophages is highly expected. AT EVs are supposed to mediate this interaction, including through straight mRNA and microRNA transfer. Thus, AT EVs from obese mice being injected intravenously are preferentially are taken up by circulating monocytes [17] and macrophages incubated with AT EVs showed adipocyte-dominant gene transcripts [18]. Another study on obese mice demonstrated that adipocytes release lipid-laden EVs that can drive macrophage migration, differentiation predominantly into M1 phenotype, activation of AT-resident macrophages, and secretion of pro-inflammatory cytokines [19,20]. One can assume that secreted AT EVs can influence monocyte/macrophage function on the arterial wall, therefore accelerating atherosclerotic processes via macrophage foam cell formation and vascular inflammation.

Macrophage foam cell formation is a central event in CVD pathogenesis. Cellular cholesterol levels depend on the balance of uptake, efflux, and endogenous cholesterol synthesis. Thereafter, an impairment of cholesterol efflux, mainly mediated via transmembrane ATP-binding cassette transporters A1 (ABCA1) and G1 (ABCG1), leads to excessive cholesterol deposition. In one recent study conducted on obese mice, visceral AT EVs were demonstrated to promote macrophage foam cell formation and M1 phenotype transition in vitro by reducing *ABCA1* expression [21]. AT EVs’ influence on cholesterol efflux, *ABCA1* and *ABCG1* gene expression as well as their transcription regulators (*PPARG*, *LXRβ (NR1H2)*, and *LXRα (NR1H3)*) in primary human monocyte-derived macrophages (MDMs) remains unknown.

The aim of this work was to evaluate the effects of EVs secreted by visceral and subcutaneous AT (VAT and SAT, respectively) from obese patients with/without T2DM on the expression of reverse cholesterol transport (RCT) related genes (*ABCA1*, *ABCG1*, *PPARG*, *LXRβ (NR1H2)*, and *LXRα (NR1H3)*) in human MDMs. RCT gene expression in VAT and SAT from each patient was assessed as well.

## 2. Results

### 2.1. Patient Clinical and Anthropometric Data

The obese cohort (n = 53) had a BMI range of 35.1 to 60.8 kg/m^2^. The average BMI was 49.6 ± 6.9 kg/m^2^ in subjects identified as obese with T2DM (n = 26), and 42.9 ± 6.4 kg/m^2^ in subjects identified as obese without T2DM (n = 27). Individuals from the control group without obesity (n = 15) had an average BMI of 25.2 ± 3.2 kg/m^2^ (19.4–29.4 kg/m^2^). Patients’ clinical and anthropometric data are presented in Table 1.

### 2.2. Characterization of AT EVs

**Figure 1:**
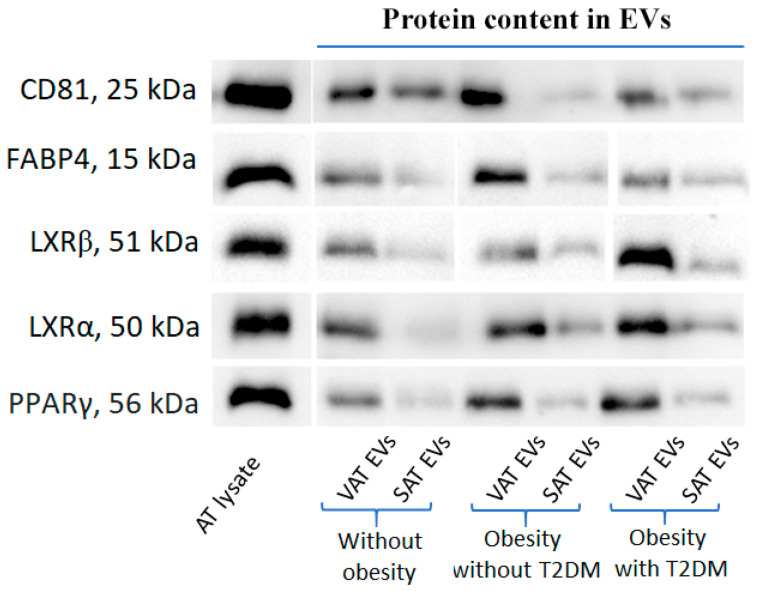
Western blot analysis of adipose tissue extracellular vesicles extracted from pooled culture mediums obtained after ex vivo cultivation of VAT and SAT of the individuals from the studied groups. Abbreviations: AT—adipose tissue, EVs—extracellular vesicles, SAT—subcutaneous AT, T2DM—type 2 diabetes mellitus, VAT—visceral AT.

For the experiments, VAT and SAT EVs were extracted from 100 mL pooled culture mediums obtained after ex vivo cultivation of VAT and SAT from the individuals from the studied groups (Table 1). The quality of AT EVs obtained via the proposed protocol of AT cultivation and subsequent EV extraction was confirmed by cryoelectron microscopy in our previous experiments [13]. In this study, all EV preparations were analyzed by an NTA to address the concentration and size distribution and a Western blot analysis for specific markers. EVs yielded a concentration in a range of 5 × 10^12^–5 × 10^13^ particles/mL with an average mode size of 85 ± 14 nm; the parameter D90 (the diameter at which 90% of the samples’ mass comprises particles with a diameter less than this value) was estimated as 185 ± 19 nm. The representative NTA of particle size and concentration of AT EVs isolated from the pooled culture medium samples of the studied groups is presented in Figure S1. NTA data were used to quantify added sample volumes of EVs in experiments with MDMs; AT EVs were added at a concentration of 10^5^ particles per cell. The isolated particles were checked for the canonical exosomal marker CD81 as well as fatty acid binding protein 4 (FABP4) as a specific marker of AT EVs (Figure 1). Additionally, AT EVs were shown to contain peroxisome proliferator-activated receptor γ (PPARγ) and liver X receptors (LXRα/β) proteins.

**Figure 2:**
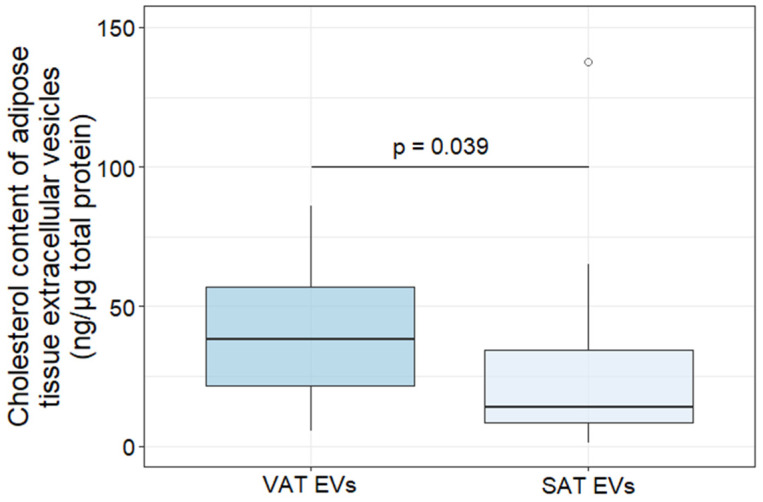
Cholesterol content of subcutaneous and visceral adipose tissue-derived extracellular vesicles of patients with obesity. Abbreviations: EVs—extracellular vesicles, SAT—subcutaneous adipose tissue, VAT—visceral adipose tissue.

The cholesterol content of AT EVs was determined in samples obtained via EV extraction from aliquoted AT culture medium samples (4 mL) from patients with obesity with/without T2DM before pooling. VAT EVs were enriched in cholesterol compared to SAT EVs in obese individuals (Figure 2). There were no differences in the cholesterol content of AT EVs depending on the T2DM status of obese patients.

### 2.3. Effects of AT EVs on RCT Gene Expression in MDMs

**Figure 3:**
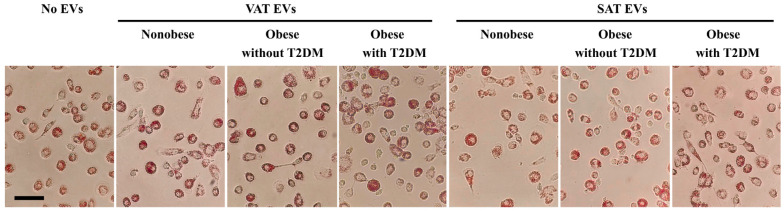
Oil Red O staining of human MDMs incubated with oxLDLs and AT EVs (scale bar, 50 μm). Abbreviations in the figure: EVs—extracellular vesicles, SAT—subcutaneous adipose tissue, T2DM—type 2 diabetes mellitus, VAT—visceral adipose tissue.

*ABCA1*, *ABCG1*, *PPARG*, *LXRα*, and *LXRβ* gene expression in individual samples of VAT and SAT in all studied groups were determined (Supplementary file: Figures S2–S4). VAT and SAT EVs were extracted from pooled samples of culture medium obtained after ex vivo cultivation of VAT and SAT of obese patients with/without T2DM and controls (Table 1). Further, *ABCA1*, *ABCG1*, *PPARG*, *LXRα*, and *LXRβ* mRNA levels were evaluated in MDMs from healthy donors treated with all types of VAT and SAT EVs for 24 h. The experiment was repeated three times for each type of AT EV; the results are presented (in percentages) as a fold-change to negative control wells (100%). The different effects of T2DM and non-T2DM obese AT EVs on RCT gene expression were demonstrated. At the same time, an incubation with oxidized low-density lipoproteins (oxLDLs) in the presence of different types of AT EVs and subsequent oil red staining did not show any differences in lipid accumulation (Figure 3).

**Figure 4:**
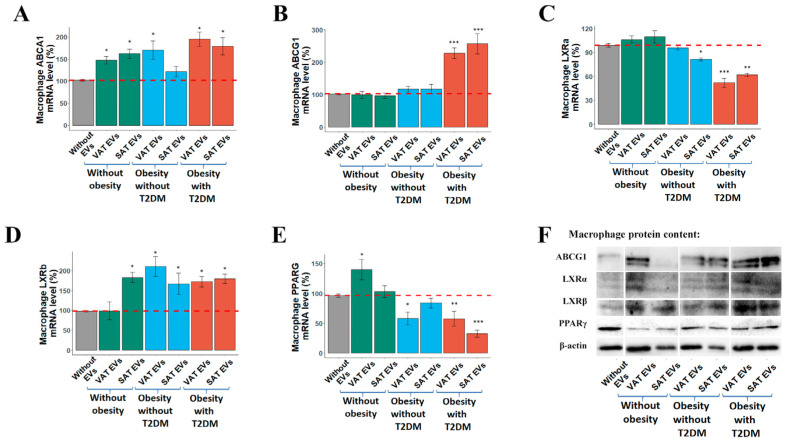
Adipose tissue extracellular vesicles dysregulate reverse cholesterol transport-related gene expression in human monocyte-derived macrophages. (**A**) *ABCA1*, (**B**) *ABCG1*, (**C**) *LXRα*, (**D**) *LXRβ*, and (**E**) *PPARG* mRNA levels in MDMs were determined by a quantitative real-time polymerase chain reaction. (**F**) Western blotting analysis of ABCG1, LXRα, LXRβ, and PPARγ in MDMs. Data are presented as the mean ± SEM. * *p* < 0.05 vs. negative control without EVs (MDMs in EV-free supplement with the addition of a negative control which was obtained from a medium that was not used for adipose tissue cultivation but was proceeded in the same ultracentrifugation cycle); ** *p* < 0.05 vs. negative control without EVs and the control group; *** *p* < 0.05 vs. negative control without EVs, the control group and obese patients without T2DM. Abbreviations in the figure: EVs—extracellular vesicles, SAT—subcutaneous adipose tissue, T2DM—type 2 diabetes mellitus, VAT—visceral adipose tissue.

*ABCG1* mRNA expression in MDMs was significantly induced by both VAT and SAT EVs of obese T2DM patients, while this effect was not observed when the EVs from AT of obese patients without T2DM or the control group were added (Figure 4B). The ABCG1 Western blot was in agreement with this pattern of *ABCG1* mRNA expression (Figure 4F). At the same time, *ABCA1* mRNA level tended to increase with the addition of all types of AT EVs, including EVs from the control group (Figure 4A). The *LXRα* mRNA level was significantly decreased both with the addition of VAT and SAT EVs from obese patients with T2DM (Figure 4C). The *LXRβ* mRNA level was increased by the addition of VAT and SAT EVs from the obese individuals regardless of T2DM manifestation (Figure 4D). A significant decrease in the *PPARG* mRNA level was observed when VAT and SAT EVs from obese patients with T2DM or VAT EVs from obese patients without T2DM were added (Figure 4E). The *PPARG* mRNA level increased with the addition of VAT EVs from the control group. LXRs and PPARγ protein levels in MDMs exposed to AT EVs did not correlate with mRNA patterns (Figure 4F). AT EVs were shown to transport PPARγ and LXRα/β proteins (Figure 1).

## 3. Discussion

Our present study is the first to investigate the effects of VAT and SAT EVs from patients with obesity and T2DM on RCT-related gene expression in primary human MDMs and shows that a T2DM diagnosis is a more important factor for defining AT EVs’ influence on MDMs. Specific *ABCG1*, *LXRα*, and *PPARG* gene expression patterns in MDMs treated with VAT and SAT EVs from patients with obesity and T2DM were demonstrated. T2DM AT EVs induced *ABCG1* gene expression whereas *LXRα* and *PPARG* mRNA levels were decreased. The *PPARG* mRNA level was decreased also when VAT EVs from obese patients without T2DM were added. In contrast, *ABCA1* and *LXRβ* mRNA levels tended to increase in MDMs with the addition of all types of AT EVs, including EVs of the control group. In adipose tissue itself during obesity, among all analyzed genes, only the *PPARG* mRNA level was reduced in both VAT and SAT.

Earlier, Barberio et al. studied the effects of VAT EVs obtained from obese humans on THP-1 macrophages [16]. Cholesterol efflux from THP-1 macrophages was significantly reduced when exposed to VAT EVs at 3 μg/mL as compared to 1 μg/mL, with no difference between incubations with VAT EVs from subjects with and without obesity. *ABCA1* and *LXRα* gene expression was firstly activated, but a higher dose of AT EVs led to a reduction with no difference between incubations with VAT EV from subjects with and without obesity. No differences were observed for the expression of the *ABCG1* and *PPARG* genes in THP-1 macrophages. These results show that the effects of AT EVs on macrophage gene expression can be dose-dependent. Still, SAT EVs as well as AT EVs in T2DM were not studied. Also, the primary MDMs used in the present study differ from THP-1 macrophages.

THP-1 cells are often used as the model to study macrophage function; however, they may act differently from primary human MDMs; cytokine profiles and bioparticle consumption were shown to be different [22]. In the case of primary MDMs, an in vivo situation is more closely reproduced. Also, in line with previous results, primary MDMs were more efficient in particle uptake compared to the THP-1 macrophages [22]. Our study was the first to analyze VAT and SAT EVs’ effects on MDMs.

Previous studies conducted on animal models investigating the potential influence of AT EVs on macrophage cholesterol efflux demonstrated complex results. Liu et al. studied EVs secreted by SAT and perivascular adipose tissue (PVAT) from wild-type C57BL/6J mice [23]. SAT EVs did not show any effects. At the same time, PVAT EVs significantly reduced foam cell formation and total cholesterol content in murine macrophages [23]. Moreover, cholesterol efflux to high-density lipoproteins (HDLs) was promoted by PVAT EVs and the upregulation of ABCA1 and ABCG1 was observed [23]. These results suggested the protective role of PVAT EVs on macrophage foam cell formation and atherosclerosis. Still, the effects of AT EVs could depend on their origin. Xie et al. demonstrated that VAT but not SAT EVs from obese mice as well as wild-type mice facilitated macrophage foam cell transformation, thus promoting the progression of atherosclerosis [21]. This effect of VAT EVs was accompanied by the downregulation of *Abca1* and *Abcg1* mRNA and protein levels. Decreased protein levels of LXRα were also observed. However, only VAT EVs from obese mice stimulated M1 polarization of macrophages and proinflammatory cytokine release; moreover, intravenous injection of VAT EVs accelerated atherosclerosis in *Apoe-/-* mice [21].

Still, results obtained from mice models could not be easily translated to humans. For example, differences in regulation of *ABCA1* expression were reported [24]. In particular, the sequence of the DR4 element (the binding site for LXRs) within a mouse *Abca1* promoter contains two replacements compared to human *ABCA1* [24]. It was shown that in mice, resident AT macrophages expressing high levels of *Abca1* do not express the transcription factor *Nr1h3* encoding LXRα, which is assumed to regulate the transcription of a large repertoire of genes linked to lipid cholesterol metabolism, including *Abca1* [25].

Based on our previous findings we could speculate that the effect of AT EVs on macrophage gene expression observed here may depend on alterations in EV content in T2DM [17]. It is worth noting that AT is a significant source of circulating microRNAs [26]. This led to the hypothesis that adipocyte-derived EV microRNAs would target mRNAs involved in macrophage cholesterol efflux and that EVs, in part, exert their pro-atherogenic effect through the transfer of these microRNAs [16]. Obesity-driven changes in AT derived EV microRNAs were previously demonstrated by us and others and could be more dramatic in T2DM [15,27,28]. However, the definite miRNA content of AT EVs in T2DM remains unclear. Insulin-signaling proteins are altered in EVs during diabetes and correlate with decreased levels of activated proteins involved in AKT signaling in insulin-resistant tissue [12]. Additionally, LXRs and PPARγ proteins could be transferred within AT EVs (Figure 1), but their influence on gene expression in recipient cells is less believable. Also, there is a need to take into account that AT EVs originate from different cell types, as was earlier illustrated by the presence of cellular markers of macrophages (Mac-2), preadipocyte (Pref-1), endothelial (VE-cadherin), or adipocyte/macrophage (aP2) wherein the PPARγ protein was detected in some subtypes of EVs [14]. At the same time, AT EVs are important for the transport of adipokines: for example, 2–10% of adiponectin in serum is associated with EVs [29]. Adiponectin has been reported to upregulate an expression of the *ABCA1* gene in human macrophages and enhance cholesterol efflux [15,30]. Lipids including cholesterol are also transported by AT EVs. Still, differences in cholesterol composition between VAT and SAT EVs in our study are not likely to explain the observed effects of EVs, in particular the similar effects of VAT and SAT EVs on patients with obesity or T2DM. The influence of other lipids, for example, fatty acids, could not be also excluded [31,32,33]. Unsaturated and saturated fatty acids were shown to differentially regulate the expression of ATP-binding cassette transporters ABCA1 and ABCG1 in human macrophages [34].

*PPARG* mRNA levels were decreased in the VAT and SAT of obese patients compared with the control group in our study (Figure S2). These results are consistent with the previous studies, demonstrating reduced *PPARG* gene expression in AT during obesity [35,36]. PPARγ is able to activate LXRs, and together, they act to inhibit the activity of proinflammatory transcription factors, including NF-κB, through direct and indirect mechanisms [37]. Additionally, LXRs are cholesterol sensors and facilitate cholesterol efflux via ABCA1 and ABCG1 transporters, thereby protecting macrophages from lipid overload and foam cell formation [38]. PPARγ activity in macrophages is associated with M2 polarization [39,40,41]. Thus, the reduction in *PPARG* gene expression in macrophages upon AT EV incubation may cause a subsequent decrease in PPARγ activity and indicate proinflammatory polarization (M1) and proatherogenic phenotype of macrophages. M1 macrophages affect the metabolic status of adipocytes by releasing cytokines (like IL-6 and TNF-α), leading to systemic glucose intolerance and insulin resistance [42]. Ying et al. found that injection of AT macrophage-derived exosomes from obese mice into lean mice reduced the expression levels of PPARγ and its target gene *Glut4* encoding glucose transporter type 4 and led to impaired insulin sensitivity [43]. Interestingly to note is that *PPARG* gene expression in human MDMs was reduced via incubation with obese AT EVs and this effect correlates with reduced gene expression in AT during obesity.

## 4. Materials and Methods

### 4.1. Study Participants

Patients with obesity with or without T2DM who underwent bariatric surgery (body mass index (BMI) > 35) were recruited for this study. A T2DM diagnosis was based on clinical and laboratory features as per the 1999 WHO criteria for diabetes classification and diagnosis [44]. Patients with the following characteristics were included: fasting plasma glucose levels ≥ 7.0 mmol/L or 2 h post-challenge glucose levels in an oral glucose tolerance test ≥ 11.1 mmol/L. The control group was formed by normoglycemic subjects without obesity and T2DM who were selected from a convenience sample of patients undergoing unrelated abdominal procedures. Adipose tissue samples for EV preparations and the study of RCT gene expression in AT were taken simultaneously during surgery.

The study protocol was in accordance with the Declaration of Helsinki and was approved by the local ethics committee of Pavlov First Saint Petersburg State Medical University, Saint Petersburg, Russian Federation (protocol 259 by 28 February 2022). Written informed consent was given by each participant.

### 4.2. Adipose Tissue Cultivation and Extraction of Extracellular Vesicles

VAT and SAT samples, collected during abdominal surgeries, were cultured using our previously published protocol [13]. Briefly, the AT samples (1–2 g) were washed with phosphate buffered saline (PBS), cut into 1–4 mm pieces, and each transferred to two 10 cm petri dishes containing 12.5 mL DMEM/F12 medium with 10% exosome-free serum (Fetal Bovine Serum, exosome-depleted, A2720803, Thermo Fisher Scientific, Waltham, MA, USA) supplemented with 1% gentamicin and incubated for 12 h at 37 °C, 5% CO_2_. The culture supernatant was prepared via serial centrifugations and filtration; specifically, it was centrifuged at +4 °C at 300× *g* for 10 min, 3500× *g* for 30 min, 10,000× *g* for 30 min, and filtered with a 0.22 syringe PES filter (Key-Labs, Qingdao, China) to remove lipids, cells, and cellular debris before ultracentrifugation. Prepared in this way, 4–5 mL aliquots of culture medium were frozen in liquid nitrogen and stored at −80 °C. A total of 100 mL of pooled culture medium (aliquots were unfrozen on ice) was diluted with PBS 1:1 and subjected to ultracentrifugation at 110,000× *g* for 2 h to pellet EVs (Optima L-90K centrifuge, Ti45 rotor, Beckman Coulter, Brea CA, USA). EVs were washed in PBS and centrifuged for the second time at 110,000× *g* for 2 h for concentration using 4 mL tubes (Optima L-90K centrifuge, SW 55Ti rotor, Beckman Coulter, Brea, CA, USA). Finally, the EV pellet was resuspended in 100 μL of PBS, and aliquots were frozen in liquid nitrogen and stored at −80 °C for further analysis. For each experiment, freshly extracted EVs were used. The negative control for further experiments was prepared from a culture medium which was not used for AT cultivation during a similar ultracentrifugation cycle.

### 4.3. Characterization Adipose Tissue Extracellular Vesicles

The size and concentration of EVs were determined by a Nanoparticle Tracking Analysis (NTA) using the NTA NanoSight LM10 analyzer, equipped with a 405 nm laser (Nano-Sight, Malvern Instruments, Malvern, UK) and a C11440-5B camera (Hamamatsu Photonics K.K., Shizuoka, Japan). Recording and data analysis were performed using the NTA software 2.3. The following parameters were evaluated during the analysis of recording monitored for 60 s: the average hydrodynamic diameter, the mode of distribution, the standard deviation, and the concentration of vesicles in the suspension. Before NTA measuring, an aliquot of the isolated EVs was thawed at room temperature and diluted with deionized water by 1000, 10,000, and 100,000 times. The measurements were performed at least three times.

The analysis of exosome-specific markers as well as AT proteins (including FABP4 as a specific marker of AT EVs [45]) was performed by a Western blot analysis. EVs were lysed 1:1 in ice-cold RIPA buffer containing 50 mMTris-HCl (pH 8.0), 150 mM NaCl, 1% Triton X-100, 0.5% sodium deoxycholate, 0.1% SDS, and a protease inhibitor cocktail (Roche, Basel, Switzerland). Protein concentrations were determined using the Micro BCA protein assay (Thermo Fisher Scientific, Waltham, MA, USA). A mass of 5 µg protein per lane was separated using 8% SDS-PAGE gels. Proteins were transferred to PVDF membranes (Millipore, Burlington, MA, USA) and pre-incubated with 5% skim milk in PBS. The blots were incubated with rabbit polyclonal, anti-FABP4 (1:1000; PA5-30591, Thermo Fisher Scientific), anti-LXRα (1:1000; ab106464, Abcam, Cambridge, UK), anti-LXRβ (1:1000, H00007376-M04, Novus Biologicals, Centennial, CO, USA), anti-PPARγ (1:1000; ab27649, Abcam), and monoclonal anti-CD81 (1:1000; ab109201, Abcam, Cambridge, UK) primary antibodies diluted in 1% skim milk in PBST (0.05% Tween 20 PBS) to prevent non-specific binding, followed by anti-rabbit HRP-conjugated secondary antibodies (1:3000; ab6721, Abcam, Cambridge, UK). Proteins were visualized using an ECL Western Blotting Detection Reagent (Thermo Fisher Scientific, Waltham, MA, USA) using the ChemiDoc Imaging system (BioRad, Hercules, CA, USA).

The cholesterol content of AT EVs was determined using an Amplex Red Cholesterol Assay Kit (Invitrogen, Thermo Fisher Scientific, Waltham, MA, USA) according to the manufacturer’s instructions, and was presented in ng per μg of total protein.

### 4.4. Human Monocyte Derived Macrophages

The mononuclear fraction was obtained from 50 mL of freshly collected whole blood from healthy donors by gradient centrifugation at 1600 rpm in Ficoll solution (Capricorn Scientific, Marburg, Germany) according to the method described previously [46]. The obtained mononuclear cells were transferred into 24-well plates in a culture medium (RPMI 1640, 2 mM L-glutamine, 10% FBS, and 1% gentamicin) at a rate of at least 5 × 10^6^ cells per well (1 mL of suspension) and incubated in a CO_2_ incubator at +37 °C for 2 h. Then, attached monocytes were cultured further in the presence of 10% autologous human serum for 5 days with a daily change of medium at an average density of 2 × 10^5^ cells per well. Cell counting was performed using an automatic cell counter TC20 (BioRad, USA). On the fifth day, a fresh culture medium containing exosome-free serum (Fetal Bovine Serum, exosome-depleted, A2720803, Thermo Fisher Scientific, Waltham, MA, USA) and isolated AT EVs at a concentration of 10^5^ particles per cell or the same volume of negative control were added to the MDMs. After 24 h incubation cells were lysed and used for RNA or protein isolation.

### 4.5. Oil Red Staining

The 5-day MDMs were incubated in a culture medium (RPMI 1640, 2 mM L-glutamine, 10% delipidated serum (Fetal Bovine Serum, lipid depleted, Biowest, Bradenton, FL, USA), and 1% gentamicin) with all types of EVs and with 50 μg/mL ox-LDL for an additional 24 h. Ox-LDLs were obtained from healthy volunteers by gradient ultracentrifugation via published protocols [47]. Then, the cells were washed 3 times with PBS and fixed in 4% paraformaldehyde for 15 min at room temperature. After that, the cells were washed with PBS and briefly with 60% isopropanol. The cells were stained with a filtered Oil Red O solution at room temperature for 20 min and then washed with 60% isopropanol. Stained cells were observed with light microscopy.

### 4.6. Extraction of RNA and qRT-PCR

RNA isolation from MDMs was performed using Qiazol reagent (Qiagen, Venlo, The Netherlands) according to the manufacturer’s instructions. A reverse transcription reaction was performed using an MMLV RT kit (Eurogen, Moscow, Russia) according to the manufacturer’s instructions. The purity of the RNA preparation was assessed by the absorbance ratio at wavelengths of 260 and 280 nm (purity criterion 2). The absence of RNA degradation was verified by electrophoresis in 1% agarose gel by the intensity ratio of bands corresponding to 28S and 18S rRNA (2:1 in the case of no degradation). The mRNA levels of *ABCA1*, *ABCG1*, *PPARG*, *LXRβ (NR1H2)*, and *LXRα (NR1H3)* genes were determined by a real-time PCR with TaqMan fluorescent probes and a PCR Master Mix (AlcorBio, Saint-Petersburg, Russia) on a CFX96 device (Biorad, Hercules, CA, USA). Threshold cycle (Ct) values were obtained and relative gene expression was normalized to two reference genes (*ACTB* and *RPLP0*). The primers and probes sequences used in this work are presented in Table S1.

### 4.7. Western Blotting for MDMs

The general details of the Western blot protocol were described in Section 4.3. MDMs were lysed in an ice-cold RIPA buffer. The lysate was centrifuged at 14,000× *g* for 15 min at 4 °C, and the supernatant was carefully aspirated into a new tube. A mass of 10 µg protein was used for the analysis. Primary antibodies used were as follows: rabbit polyclonal ABCG1 (1:750; alm505149, Almabion, Voronezh, Russia), anti-LXRα (1:1000; ab106464, Abcam, Cambridge, UK), anti-LXRβ (1:1000, H00007376-M04, Novus Biologicals, Centennial, CO, USA), anti-PPARγ (1:1000; ab27649, Abcam), and anti-β-actin (1:10,000; ab8227, Abcam, Cambridge, UK).

### 4.8. Statistical Analysis

The conformity of findings to normal distribution was tested using the Shapiro–Wilk test for non-normally distributed variables, comparisons between two groups were performed using the Mann–Whitney U test, while those among three groups were performed using the Kruskal–Wallis and Dunn’s post hoc tests. Correlations between variables were tested by using a two-tailed Spearman’s test. The level of significance was set at *p* < 0.05. A statistical analysis was performed using R Studio (R version 4.3.1 (2023-06-16 ucrt), RStudio version 2023.6.1.524, PBC).

## 5. Conclusions

In conclusion, the T2DM status of obese patients influenced RCT gene expression in primary human MDMs under AT EV treatment independently of EV origin (VAT or SAT EVs), leading to the upregulation of *ABCG1* and the downregulation of *LXRα* and *PPARG* gene expression. Thus, the T2DM status of obese patients had a more profound influence on AT EVs’ effect on RCT gene expression in MDMs than obesity alone.

## Figures and Tables

**Table 1:** Baseline demographic, clinical, and biochemical data of patients.

Studied Groups	Obesity without Type 2 Diabetes Mellitus N = 27	Obesity with Type 2 Diabetes Mellitus N = 26	Control Group N = 15	*p*
Age	41.7 ± 11.3	44.4 ± 10.8	47.0 ± 13.5	^1^ 0.476 ^2^ 0.721 ^3^ 0.974
Sex (male/female)	6/21	7/19	4/11	
Body mass index, kg/m^2^	42.9 ± 6.4	49.6 ± 6.9	25.2 ± 3.2	**^1^ 0.027** **^2^ 0.000** **^3^ 0.000**
Weight, kg	121.1 ± 18.0	137.5 ± 23.3	74.5 ± 11.9	^1^ 0.099 **^2^ 0.001** **^3^ 0.000**
Waist circumference, cm	120.2 ± 14.3	139 ± 16.0	nd	**^1^ 0.006**
Hip, cm	129.4 ± 13.8	134.6 ± 16.2	nd	^1^ 0.361
Waist-to-hip ratio	0.9 ± 0.1	1.0 ± 0.1	nd	**^1^ 0.037**
Glucose, nmol/L	5.4 (4.3–8.1)	7.3 (5.4–14.9)	5 (4.3–6.6)	**^1^ 0.000** ^2^ 0.700 **^3^ 0.000**
Insulin, µIU/mL	14.3 (9.4–41.4)	26.6 (8.7–79.4)	nd	**^1^ 0.035**
HOMA-IR index	3.5 (1.8–10.8)	8.8 (4.2–23.4)	nd	**^1^ 0.004**
C-peptide. ng/mL	2.7 (1.8–4.6)	3.9 (1.9–11.9)	nd	**^1^ 0.014**
HbA1c, %	5.5 (5.1–6.0)	6.8 (5–11.9)	nd	**^1^ 0.000**
Total cholesterol, mmol/L	4.9 ± 1.1	5.1 ± 0.9	nd	^1^ 0.529
HDL, mmol/L	1.4 ± 0.3	1.2 ± 0.2	nd	^1^ 0.383
LDL, mmol/L	2.8 ± 1.0	2.7 ± 0.8	nd	^1^ 0.897
Triglycerides, mmol/L	1.3 (0.6–4.3)	2.0 (0.9–4.4)	nd	**^1^ 0.022**

Notes: nd—not determined; ^1^ non-T2DM vs. obese T2DM, ^2^ non-T2DM obese vs. control, ^3^ T2DM vs. control. Abbreviations: HOMA-IR—homeostasis model assessment of insulin resistance index, HDL—high-density lipoproteins, LDL—low-density lipoproteins. Clinical and experimental data are presented as the mean ± the standard deviation (SD) and the median (min–max) depending on the distribution.

## Data Availability

Data are contained within the article and Supplementary Materials.

## Supplementary Materials

The following supporting information can be downloaded at: https://www.mdpi.com/article/10.3390/ijms25126457/s1, references [25,48,49,50,51,52,53] are cited in Supplementary Materials.
