# Supplementary material for: Extracellular Vesicles Secreted by Adipose Tissue during Obesity and Type 2 Diabetes Mellitus Influence Reverse Cholesterol Transport-Related Gene Expression in Human Macrophages
Source: Int J Mol Sci. 2024 Jun 12;25(12):6457. doi: 10.3390/ijms25126457 (PMC11204239; doi:10.3390/ijms25126457)
Supplement: Supplementary file 1 [file ijms-25-06457-s001.zip › ijms-3009599-supplementary.pdf]

SUPPLEMENTAL MATERIALS

for

Extracellular vesicles secreted by adipose tissue during obesity and type 2 diabetes mellitus influence reverse cholesterol transport gene expression in human macrophages

Kseniia V. Dracheva[1,2], Irina A. Pobozheva[1,2], Kristina A. Anisimova[2], Aleksandra A. Panteleeva[1,2], Luiza A. Garaeva[1], Stanislav G. Balandov[2], Zarina M. Hamid[2], Dmitriy I. Vasilevsky[2], Sofya N. Pchelina[1,2,3], Valentina V. Miroshnikova[1,2*]

[1]Petersburg Nuclear Physics Institute named by B.P. Konstantinov of National Research Centre “Kurchatov Institute”, Gatchina, Russian Federation

[2]Pavlov First Saint Petersburg State Medical University, St.-Petersburg, Russian Federation

[3]Federal State Budgetary Research Institution "Institute of Experimental Medicine", St.-Petersburg, Russian Federation

* Correspondence: miroshnikova_vv@pnpi.nrcki.ru, v.v.mirosh@gmail.com

Nanoparticle tracking analysis (NTA)

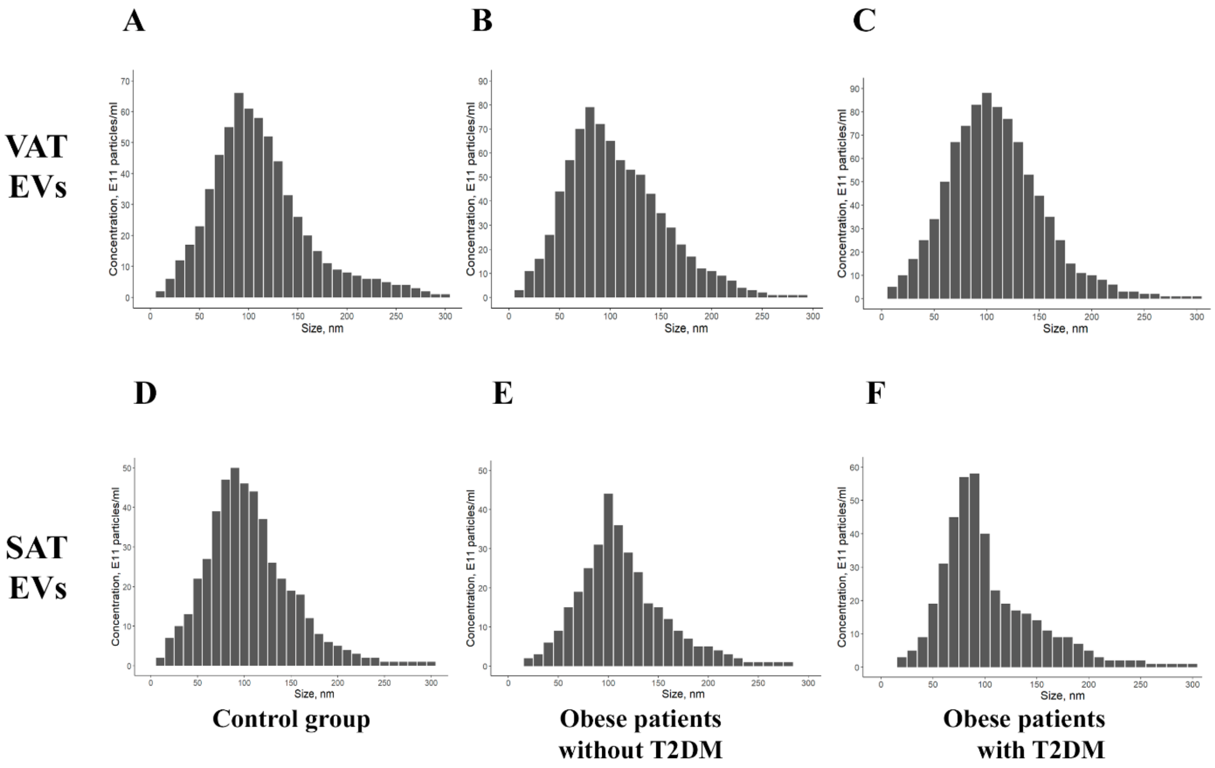

Figure S1. NTA of particle size and concentration adipose tissue EVs isolated from pooled culture medium samples: A – visceral adipose tissue, the control group; B - visceral adipose tissue, obese patients without type 2 diabetes; C - visceral adipose tissue, obese patients with type 2 diabetes; D – subcutaneous adipose tissue, the control group; E - subcutaneous adipose tissue, obese patients without type 2 diabetes; F - subcutaneous adipose tissue, obese patients with type 2 diabetes.

RCT gene expression in adipose tissue

ABCA1, *ABCG1*, *PPARG*, *LXRβ*, *LXRα* gene expression was evaluated in SAT and VAT samples in the studied groups (Table 1 of the main manuscript) via real-time PCR.

RNA isolation from SAT and VAT samples was performed using Qiazol reagent (Qiagen, Netherlands) with subsequent removal of genomic DNA impurity by DNase treatment (DNase I, RNase-free, Thermo Fisher Scientific, USA) according to the manufacturer's instructions. Reverse transcription reaction was performed using the RevertAid First Strand cDNA (Thermo Scientific, USA) according to the manufacturer's instructions. The purity of the RNA preparation was assessed by the absorbance ratio at wavelengths of 260 and 280 nm (purity criterion 2). The absence of RNA degradation was verified by electrophoresis in 1% agarose gel by the intensity ratio of bands corresponding to 28S and 18S rRNA (2:1 in the case of no degradation). mRNA levels of *ABCA1, ABCG1, PPARG, LXRβ (NR1H2), LXRα (NR1H3)* genes were determined by real-time PCR with TaqMan fluorescent probes and PCR Master Mix (AlcorBio, Russia) on the CFX96 device (Biorad, USA). Threshold cycle (Ct) values were obtained and relative gene expression was normalized to two reference genes (*ACTB* and *RPLP0*). The primers and probes sequences used in this work are presented in the Table S1.

Table S1. Primers and probes used in the work.

Gene	Sequencing (5' → 3') of forward and reverse primers and probe
ABCA1<br>(target gene)	5'-CTCCTGTGGTGTTTCTGGATG-3'<br>5'-CTTGACAACACTTAGGGCACAA-3'<br>5' (FAM)-AAGCCCGGCGGTTCTTGTGG -3'(RTQ1)
ABCG1<br>(target gene)	5'-CACGTACCTACAGTGGATGT-3'<br>5'-GTCTAAGCCATAGATGGAGA-3'<br>5' (FAM)-CTATGTCAGGTATGGGTTCGAAG-3'(RTQ1)
LXRα (NR1H3)<br>(target gene)	5’ -TCACCTTCCTCAAGGATTTCA-3'<br>5’ -TCGAAGATGGGGTTGATGA -3'<br>5’(ROX)-TAACCGGGAAGACTTTGCCAAAGCA-3’ (RTQ2)
LXRβ (NR1H2)<br>(target gene)	5'-CTGTTGCTTGGAGAGGGGC-3'<br>5'-CGTGGTAGGAGAGGACATGG-3'<br>5'(FAM)-CTGGAGAGAGGCTGCTCCGTGA-3'(RTQ1)
PPARG<br>(target gene)	5′-GATGTCTCATAATGCCATCACGTT-3'<br>5'-GGATTCAGCTGGTCGATATCACT-3'<br>5'(FAM)-CCAACAGCTTCTCCTTCTCGGCCTG-3'(RTQ1)
ACTB<br>(reference gene)	5'-CGTGCTGCTGACCGAGG-3'<br>5'-ACAGCCTGGATAGCAACGTACA-3'<br>5'(R6G)-CCAACCGCGAGAAGATGACCCAGAT-3'(BHQ1)
RPLPO<br>(reference gene)	5'-GATCAGGGACATGTTGCTGG-3'<br>5'-GACTTCACATGGGGCAATGG-3'<br>5'(ROX)-CAATAAGGTGCCAGCTGCTGC-3'(RTQ2)

SAT and VAT *PPARG* mRNA levels were reduced in obese patients compared with the control group regardless of T2DM diagnosis (Figure S1). SAT *PPARG* mRNA levels negatively correlated with plasma levels of glucose (r=-0.401, p=0.002), C-peptid (r=-0.354, p=0.040), glycated hemoglobin (r=-0.311, p=0.05), triglycerides (r=-0.497, p=0.001), HOMA-IR (r=-0.398, p=0.020).

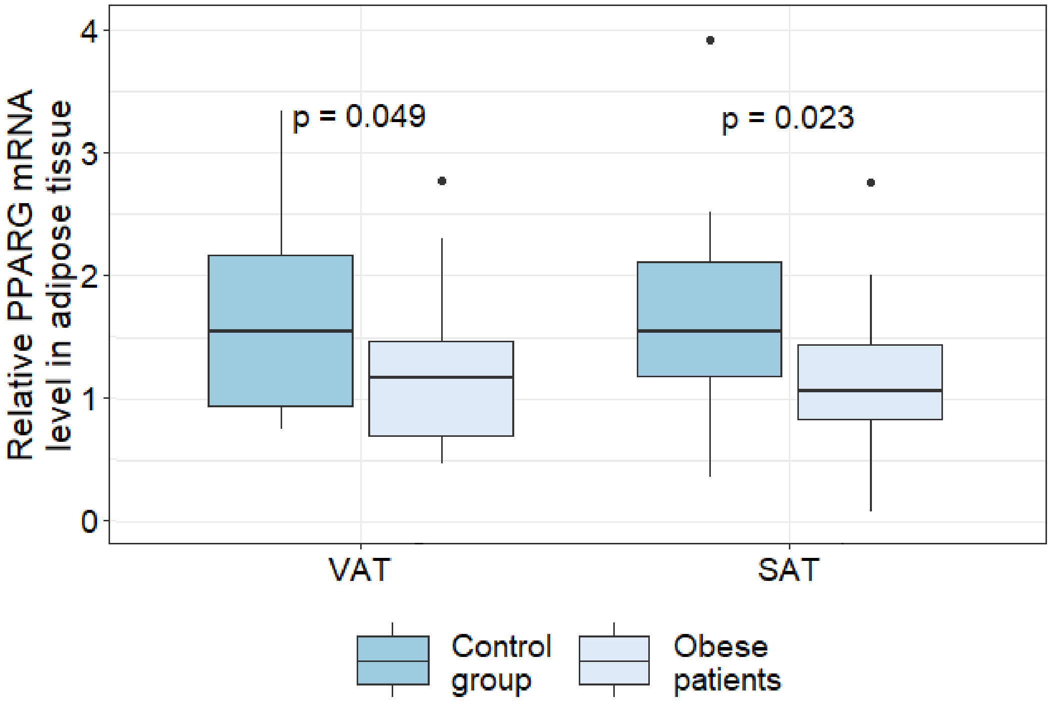

Figure S2. *PPARG* mRNA levels in subcutaneous and visceral adipose tissue in combined obese cohort and nonobese controls.

ABCA1 mRNA levels were reduced in morbidly obese without T2DM compared with the control group (p = 0.029) (Figure S2). *ABCG1* mRNA did not differ between studied groups (Figure S2) as well as *LXRα/β* (Figure S3).

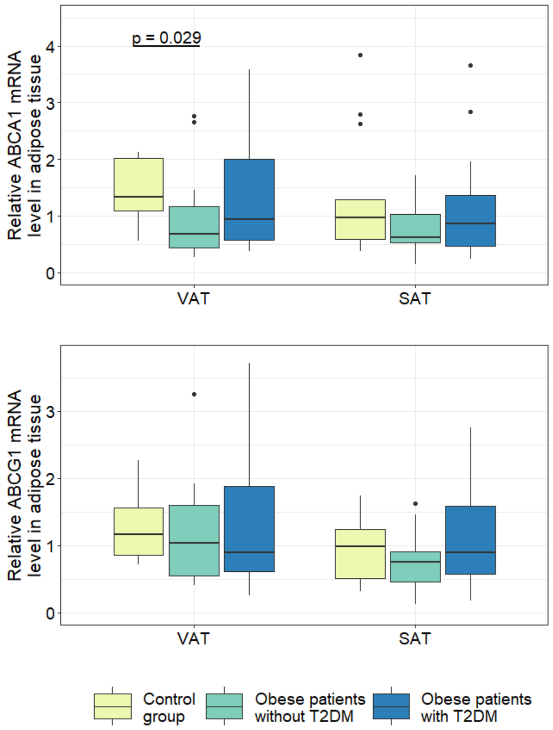

Figure S3. *ABCA1* and *ABCG1* mRNA levels in subcutaneous and visceral adipose tissue in the studied groups.

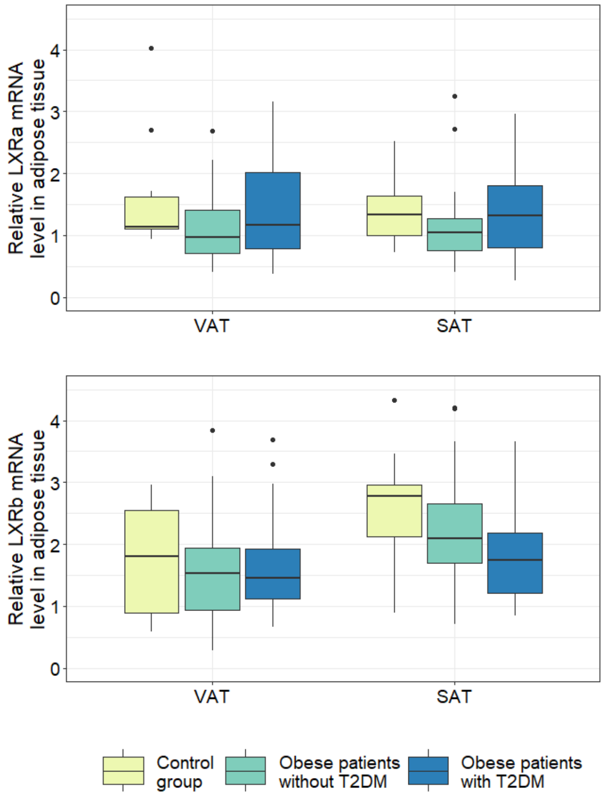

Figure S4. *LXRa* and *LXRb* mRNA levels in subcutaneous and visceral adipose tissue in the studied groups.

ABCA1 takes part in HDL biogenesis by facilitating the flow of free cholesterol and phospholipids to extracellular lipid-free apolipoprotein A-I, forming nascent HDL particles [48]. ABCG1 function is suspected as cholesterol redistribution to cell-surface domains where it becomes available for HDLs [48-50]. Our results are consistent with data obtained by Vincent et al: morbidly obese patients with insulin resistance without T2DM manifestation undergoing bariatric surgery had lower *ABCA1* mRNA and protein levels in VAT compared with the control group [48]. However, previous studies supposed that *ABCA1* and *ABCG*1 gene expression in AT could be depended on many factors as age, sex, degree of obesity, insulin sensitivity and even smoking status [48, 51-53]. It is interesting to note that *ABCA1* and *ABCG1* mRNA levels in SAT and VAT of patients with T2DM are very variable.

Interestingly, *ABCA1* and *ABCG1* mRNA levels in MDMs incubated in the presence AT EVs in our study also were not correlated. *ABCG1* mRNA increased when MDMs were incubated with AT EVs of patients with obesity and T2DM and this effect was not depended on fat depot. Both SAT and VAT AT EVs of obese diabetic patients stimulated *ABCG1* expression. *ABCA1* tended to increase in MDMs with the addition of all types of AT EVs including VAT EVs of the control group. This effect cholesterol rich EVs on the *ABCA1* expression could be linked with the fact that resident AT macrophages unlike other tissues play a role in the regulation of post-prandial HDL [25].

48. Vincent V, Thakkar H, Aggarwal S, Mridha AR, Ramakrishnan L, Singh A. ATP-binding cassette transporter A1 (ABCA1) expression in adipose tissue and its modulation with insulin resistance in obesity. *Diabetes Metab Syndr Obes*. Published online 2019:275-284.

49. Yvan-Charvet L, Wang N, Tall AR. Role of HDL, ABCA1, and ABCG1 transporters in cholesterol efflux and immune responses. *Arterioscler Thromb Vasc Biol*. 2010;30(2):139-143.

50. Vaughan AM, Oram JF. ABCG1 redistributes cell cholesterol to domains removable by high density lipoprotein but not by lipid-depleted apolipoproteins. *Journal of Biological Chemistry*. 2005;280(34):30150-30157.

51. Miroshnikova V V., Panteleeva AA, Pobozheva IA, et al. ABCA1 and ABCG1 DNA methylation in epicardial adipose tissue of patients with coronary artery disease. *BMC Cardiovasc Disord*. 2021;21(1):566. doi:10.1186/s12872-021-02379-7

52. Panteleeva AA, Razgildina ND, Brovin DL, et al. The Expression of Genes Encoding ABCA1 and ABCG1 Transporters and PPARγ, LXRβ, and RORα Transcriptional Factors in Subcutaneous and Visceral Adipose Tissue in Women with Metabolic Syndrome. *Mol Biol*. 2021;55(1):56-65. doi:10.1134/S0026893321010131

53. Miroshnikova VV, Panteleeva AA, Bazhenova EA, et al. Regulation of ABCA1 and ABCG1 gene expression in the intraabdominal adipose tissue. *Biomeditsinskaya Khimiya*. 2016;62(3):283-289. doi:10.18097/PBMC20166203283
